# Editorial: Catalytic materials and processes for a low-carbon future

**DOI:** 10.3389/fchem.2023.1156434

**Published:** 2023-02-16

**Authors:** Jesus Gandara-Loe, Tomas Ramirez Reina, Laura Pastor-Peréz

**Affiliations:** ^1^ CMACs, KU Leuven, Leuven, Belgium; ^2^ Inorganic Chemistry Department and Materials Science Institute, University of Seville-CSIC, Sevilla, Spain

**Keywords:** CO_2_ valorisation, CO_2_ capture, heterogeneous catalysts, homogeneous catalysis, catalyst design

Catalysis is a core area of contemporary science posing major fundamental and conceptual challenges while being at the heart of the chemical industry—an immensely successful and important part of the overall global economy. In the context of global challenges, a new generation of catalytic processes and advanced catalytic materials are needed to achieve net zero emissions and fight global warming. In this sense, carbon capture and reutilization is a key step to reaching a low-carbon future through CO_2_ conversion into added-value chemicals or fuels. Presently, carbon capture at the industrial level is based on aqueous amine scrubbing. However, other technologies such as adsorption in porous materials such as zeolites, metal-organic frameworks (MOFs), or silicas have been reported as a promising route for CO_2_ sequestration (Gandara-Loe et al., 2021). Furthermore, CO_2_ conversion has been explored using different catalytic systems to obtain added-value chemicals or fuels such as syngas, methanol, acetic acid, ethanol, olefins, C2 chemical, or other fuels (Centi and Perathoner, 2022; Yang et al., 2022).

As mentioned before, CO_2_ can be directly or indirectly (*via* syngas) hydrogenated to added-value molecules such as hydrocarbons which can only be achieved by an in-deep catalyst design involving physicochemical aspects such as reaction kinetics, and selectivity and also catalyst stability and regenerability.

Based on this, the Research Topic inaugurates our new section on Catalytic Reactions and Chemistry by celebrating the success of catalytic technologies as essential enablers in pursuing the transition toward sustainable societies.

The spirit of this Research Topic is to showcase successful stories in the field of catalysis as cornerstone to drive social wellbeing. Being one of the most interdisciplinary fields, catalysis plays a critical role in environmental protection and industrial development. Nowadays, due to the consecration of highly-qualified researchers, in addition to the strong collaboration, industry-academy catalysis represents a central technology to achieve the transition towards a low-carbon future.

This Research Topic counts with four research articles and one review that encompasses the advances in the design, synthesis, characterization, and optimisation of catalysts for sustainability and energy applications, and CO_2_ valorisation.

In this sense, (Cui et al.) presented an in-depth review summarizing the advances in the catalytic hydrogenation of carbon dioxide which evidence the conversion of what is considered waste into added-value hydrocarbons. This review discusses different routes for CO_2_ hydrogenation to hydrocarbon including light olefins, fuels oils such as gasoline or jet fuel, and aromatics compounds, having a special emphasis on catalyst development. Finally, it is summarized the research challenges and opportunities associated with this reaction.

The design of catalysts for heterogeneous reactions is the main topic in the fourth research article included in this Research Topic. For instance, the design of highly active Ni catalysts supported on carbon nanofibers was explored by (Frecha et al.) for the hydrolytic hydrogenation of cellobiose. It was observed that by varying the impregnation technique it was possible to obtain carbon-nanofiber-supported catalysts with a wide range of Ni crystal sizes. This link between the particle size and the reactivity made it possible to establish a compromise between performance and resistivity to the metal active phase.

Additionally, (Puello-Polo et al.) Contributed to the Research Topic with a study related to the design of Ni, Mo and Ni-Mo mixed with phosphidic - sulfonic phase supported in Al_2_O_3_-MgO catalysts for green diesel production *via* hydrotreating of stearic and oleic acids. It was observed that mixed phosponic—sulphidic species acted as structural promoters in the generation of larger chain hydrocarbons such as heptadecane and octadecane.

As biobutanol has been targeted as an alternative to renewable sources that mitigate climate change, (Portillo Crespo et al.) contributed with a research article giving insights on the Guebert reaction, which is used for the production of biobutanol from bioethanol. The authors explored and optimized the reaction parameters using Mg-Al spinel-type catalyst. The catalyst demonstrated exceptional performance in the Guebert reaction with a promising activity/butanol selectivity balance, but also long-term stability.

Finally, the articles included in this Research Topic not only encompass the design of catalysts for the conversion and upgrading of compounds into added-value chemicals but also the design of catalysts for environmental remediation. In this sense, (Tagar et al.) reported the synthesis of highly ordered CaO obtained from the calcination of cuttlefish bone and its application as an alkali catalyst for the degradation of methylene blue from water.

As has been mentioned in this editorial, The spirit of this Research Topic is to showcase successful stories in the field of catalysis as cornerstone to drive social wellbeing. Being one of the most interdisciplinary fields, catalysis plays a critical role in environmental protection and industrial development. Nowadays, due to the consecration of highly-qualified researchers, in addition to the strong collaboration, industry-academy catalysis represents a central technology to achieve the transition towards a low-carbon future. The five manuscripts included are a great representation of the quality and relevance of the contributions to new approaches to combat global warming, paving the way towards a modern sustainable society.
